# Supplementary material for: 2-iminobiotin, a selective inhibitor of nitric oxide synthase, improves memory and learning in a rat model after four vessel occlusion, mimicking cardiac arrest
Source: PLoS One. 2023 Sep 25;18(9):e0291915. doi: 10.1371/journal.pone.0291915 (PMC10519591; doi:10.1371/journal.pone.0291915)
Supplement: S1 Table — Surviving cells in the CA1 region of the hippocampus at the left and right side for all treatment groups. * p = 0.002 one-way ANOVA, #sham versus all treatment groups p<0.020 Dunnett’s post hoc analysis. (DOCX) [file pone.0291915.s001.docx]

| Region  Treatment | CA1  left | CA1  right |
| --- | --- | --- |
| sham | 159±21*^#^ | 201±25*^#^ |
| vehicle | 40±11 | 38±12 |
| 1.1 mg/kg | 22±7 | 36±10 |
| 3.3 mg/kg | 81±18 | 61±15 |
| 10 mg/kg | 73±14 | 53±15 |
| 30 mg/kg | 49±14 | 50±13 |
