# Supplementary material for: 2-iminobiotin, a selective inhibitor of nitric oxide synthase, improves memory and learning in a rat model after four vessel occlusion, mimicking cardiac arrest
Source: PLoS One. 2023 Sep 25;18(9):e0291915. doi: 10.1371/journal.pone.0291915 (PMC10519591; doi:10.1371/journal.pone.0291915)
Supplement: Attachment — Submitted filename: Response to reviewers.docx [file pone.0291915.s002.docx]

**REBUTTAL LETTER: Response to reviewers Plos One**

**PONE-D-22-32387**

**2-Iminobiotin, a selective inhibitor of nitric oxide synthase, improves memory and learning in a rat model after four vessel occlusion, mimicking cardiac arrest.**

Thank you very much for carefully reviewing the manuscript and giving the opportunity to improve the manuscript.

**Reviewer #1**: This is interesting manuscript titled "2-Iminobiotin, a selective inhibitor of nitric oxide synthase, improves memory and learning in a rat model after four vessel occlusion, mimicking cardiac arrest."
The authors should discuss in more detail how selective 2-Iminobiotin is and for what type of nitric oxide synthase.

*2-Iminobiotin is an inhibitor of nitric oxide synthase (NOS), but it inhibits the 3 different isoforms (endothelial, neuronal and inducible NOS) in different ways. In in vitro studies,* *2-IB, which has a similar chemical structure as L-Arg exhibited selective inhibition of neuronal and inducible nitric oxide synthase activity of murine iNOS and rat cNOS with no effect on endothelial nitric oxide synthase (eNOS) (Sup SJ, 1994). Additionally, radioligand binding assays were performed by Ricerca Biosciences LLC, Taipei, Taiwan for 174 enzymes including all NOS enzymes (Kun-Yuan Lin, 2010). Inhibitory effects of 2-IB were identified against all NOS isoenzymes, but the IC50 was much lower for iNOS and nNOS, than for eNOS, suggesting a more powerful inhibition of inducible and neuronal NOS than endothelial NOS (see Table below).*

| **Biochemical assay** | **Species** | **Concentration** | **% Inhibition** | **IC50** |
| --- | --- | --- | --- | --- |
| Nitric oxide synthase, neuronal (nNOS) | Rat | 300 µM | 68 | 142 µM |
| Nitric oxide synthase, endothelial (eNOS) | Bovine | 1000 µM | 63 | 646 µM |
| Nitric oxide synthase, inducible (iNOS) | Mouse | 300 µM | 81 | 96 µM |

*In the discussion lines 307-313 are added to describe the above with the mentioning of 2 references (35,36].*

**Reviewer #2:** Line 28 - s.c. abbreviation missing. *Is added*
Line 32 - VO abbreviation missing. *Is written full out now*
Line 98-100 – References missing in methodology section. *Lines 98-100 are describing in short the methodology, named in reference 14*. This was cleared up by adding the words, “in short”.
Line 134-135- How are the doses designed and the significance of these dosage regimen. The Pharmacokinetic and pharmacodynamic data about the drug is not mentioned. *We agree with the reviewer that this needs more explanation. Based on earlier studies with 2-iminobiotin in newborn rats, an optimal dose of 10 mg/kg/dose was found, when given in 3 gifts s.c. (directly upon reperfusion and at 12 and 24h after reperfusion). So for this study, we chose a dose regimen with a (3 times) lower and higher treatment schedule to determine the optimal dosage in adult rats after reperfusion. The optimal dosage was added to the methods (ref. 15).*
Line 141 & 189 - empty lines. *Lines are deleted as well as line 173 and 171*
Line 127 – Terminated --- Scarified would be a better and more suitable word. *Terminated has been replaced by sacrified.*
Line 210 - Parametric References are missing. *In sec is added.*
Line 230 – Global hypoxic-ischemia – occlusion duration is 15 seconds and the clips on the MCA would be at different time interval. So due to this CA1 regional and histology should be shared and demonstrated even when the difference was not seen. *We added a table as Supporting Information File to show the results.*
Line 233 – CA1 region histology missing for 1.1 dosage. *For a representative example only the best dosage (3.3 mg/kg/dose) was chosen to depict, the 1.1, 10 and 30 mg/kg/dose are not displayed.*
Line 80 – Only female rats are used for this experiment and the relationship between the hormonal cycle is not studied in this study. Hormonal relationship between the 2-IB is missing as well. *Aim of this study was not to study the hormonal relationship between 2-IB and HI reperfusion injury. But we agree with the reviewer, that this is an important question for translation to clinical studies. This was already described in lines 274-275.*
Line 310 – Why its is mentioned as 12hrs for 24hrs, the dosage was administered at 12hrs interval for 24hrs. *We agree that this is confusing and changed it to “for 3 gifts”*

Line 315 – If the IV bolus dosage of 0.055-0.165mg/kg/dose was demonstrated then why was the regimen set for 10 & 30 mg dose rather the dosage to be considered should be within the +-10-15% variability region. Any justification should be mentioned(missing references). *Currently, the clinical phase2a study is published, which explains the PK rationale. We made a reference to this article [Admiraal MM, Velseboer DC, Tjabbes H, Vis P, Peeters-Scholte C, Horn J. Neuroprotection after cardiac arrest with 2-iminobiotin: a single center phase IIa study on safety, tolerability, and pharmacokinetics. Front Neurol. 2023 Jun 2;14:1136046. doi: 10.3389/fneur.2023.1136046].*

**PONE-D-22-32387**

Journal requirements:

When submitting your revision, we need you to address these additional requirements.

1. Please ensure that your manuscript meets PLOS ONE's style requirements, including those for file naming. The PLOS ONE style templates can be found at

<https://journals.plos.org/plosone/s/file?id=wjVg/PLOSOne_formatting_sample_main_body.pdf> and

[https://journals.plos.org/plosone/s/file?id=ba62/PLOSOne_formatting_sample_title_authors_affiliations.pdf](https://eur03.safelinks.protection.outlook.com/?url=https%3A%2F%2Fjournals.plos.org%2Fplosone%2Fs%2Ffile%3Fid%3Dba62%2FPLOSOne_formatting_sample_title_authors_affiliations.pdf&data=05%7C01%7Cc.m.p.c.d.peeters-scholte%40lumc.nl%7Cc8d08749c6484a144bfa08db9ff65f62%7Cc4048c4fdd544cbd80495457aacd2fb8%7C0%7C0%7C638279652887175723%7CUnknown%7CTWFpbGZsb3d8eyJWIjoiMC4wLjAwMDAiLCJQIjoiV2luMzIiLCJBTiI6Ik1haWwiLCJXVCI6Mn0%3D%7C3000%7C%7C%7C&sdata=u4qluDVxySftd8vAB5rlu9D5EK2T6FMOUBeKLmnAmh0%3D&reserved=0).

*As recommended file names were used as provided in the PLOS ONE’s requirements.*

2. We note that the grant information you provided in the ‘Funding Information’ and ‘Financial Disclosure’ sections do not match.

When you resubmit, please ensure that you provide the correct grant numbers for the awards you received for your study in the ‘Funding Information’ section.

*We have not received any grants for this research. Neurophyxia B.V. has paid MD Biosciences, the CRO, to perform this trial, which is described in the current manuscript. This is stated in the Financial Disclosures.*

3. We note that you have a patent relating to material pertinent to this article. Please provide an amended statement of Competing Interests to declare this patent (with details including name and number), along with any other relevant declarations relating to employment, consultancy, patents, products in development or modified products etc. Please confirm that this does not alter your adherence to all PLOS ONE policies on sharing data and materials, as detailed online in our guide for authors <http://journals.plos.org/plosone/s/competing-interests> by including the following statement: "This does not alter our adherence to  PLOS ONE policies on sharing data and materials.” If there are restrictions on sharing of data and/or materials, please state these. Please note that we cannot proceed with consideration of your article until this information has been declared.

This information should be included in your cover letter; we will change the online submission form on your behalf.

4. We note that you have included the phrase “data not shown” in your manuscript. Unfortunately, this does not meet our data sharing requirements. PLOS does not permit references to inaccessible data. We require that authors provide all relevant data within the paper, Supporting Information files, or in an acceptable, public repository. Please add a citation to support this phrase or upload the data that corresponds with these findings to a stable repository (such as Figshare or Dryad) and provide and URLs, DOIs, or accession numbers that may be used to access these data. Or, if the data are not a core part of the research being presented in your study, we ask that you remove the phrase that refers to these data.

*Data are shown now as a Supporting Information file (S1 Table).*

5. Please review your reference list to ensure that it is complete and correct. If you have cited papers that have been retracted, please include the rationale for doing so in the manuscript text, or remove these references and replace them with relevant current references. Any changes to the reference list should be mentioned in the rebuttal letter that accompanies your revised manuscript. If you need to cite a retracted article, indicate the article’s retracted status in the References list and also include a citation and full reference for the retraction notice.

*In the rebuttal letter all added references are named. No references have been retracted.*

Additional Editor Comments (if provided):

The authors should perform additional experiments to demonstrate how selective 2-Iminobiotin is and for what type of nitric oxide synthase.

*Results on selectivity of NOS inhibition are now added to the discussion with reference to 2 earlier studies that investigated the selectivity of NOS [35,36].*
